# A Comprehensive Biophysical Analysis of the Effect of DNA Binding Drugs on Protamine-induced DNA Condensation

**DOI:** 10.1038/s41598-019-41975-8

**Published:** 2019-04-10

**Authors:** Sakshi Gupta, Neha Tiwari, Manoj Munde

**Affiliations:** School of Physical Sciences, Jawaharlal Nehru University, New Delhi, 110067 India

## Abstract

DNA condensation is a ubiquitous phenomenon in biology, yet the physical basis for it has remained elusive. Here, we have explored the mechanism of DNA condensation through the protamine-DNA interaction, and by examining on it the influence of DNA binding drugs. We observed that the DNA condensation is accompanied by B to Ψ-DNA transition as a result of DNA base pair distortions due to protamine binding, bringing about the formation of toroidal structure through coil-globule transition. The binding energetics suggested that electrostatic energy, bending energy and hydration energy must play crucial roles in DNA condensation. EtBr intercalation interferes with the protamine-DNA interaction, challenging the distortion of the DNA helix and separation of DNA base pairs by protamine. Thus, EtBr, by competing directly with protamine, resists the phenomenon of DNA condensation. On the contrary, netropsin impedes the DNA condensation by an allosteric mechanism, by resisting the probable DNA major groove bending by protamine. In summary, we demonstrate that drugs with distinct binding modes use different mechanism to interfere with DNA condensation.

## Introduction

The DNA of a living organism is condensed in a compact form in order to store, transport and preserve the genetic material. Naturally occurring polyamines, lipids or proteins are known to drive DNA condensation inside cells^1–9^. A packaged DNA allows accessibility to biological ligands, when required, influencing essential processes like transcription, replication, repair, and recombination^10^. Drugs with clinical significance also need to access specific gene sequences embedded in the compact DNA in order to realize their therapeutic role. How these drugs affect the DNA condensed states will have great implications in how they regulate biological processes. In view of this, it is first essential to understand the mechanism of DNA condensation. Although several studies have been done, the topic is still the subject of intense scrutiny due to lack of in-depth rationalization pertaining to the mechanism of DNA condensation. Also, since DNA condensation has direct applicability in DNA formulation as a part of gene delivery systems, it has drawn huge attention^11^.

Several agents have been used to study *in-vitro* DNA condensation^2–6,8,12–14^. Protamines are small basic proteins that are known to induce the most compact states of DNA in the sperm cells^15^. Such compact states can serve as simple models to investigate the properties of more complex compact states of DNA *in vivo*. DNA is a highly charged and very stiff polymer. Also, it offers wide variety of ligand-binding sites such as intercalation, major groove, minor groove, phosphate backbone etc. Understanding how these various DNA features are exploited by protamine in DNA condensation is a key to unraveling the mechanism of DNA condensation. Previous studies have mostly focused on qualitative understanding of the protamine-DNA interaction^15–20^. Also, due to the absence of a crystal or solution structure, the precise binding mode of protamine and location of Arg residues on the DNA helix remain ambiguous. The importance of Arg in the protamine induced DNA condensation was first recognized by investigating the effect of the neutral and negatively charged amino acids^16^. These results are well supported by recent experiments on the physical basis of why arginines are preferred over lysines in a protamine sequence^17^. The role of electrostatic interaction has also been proposed since long, however, the detailed energetic parameters are not known. Altogether, the precise nature of the interaction responsible for the DNA condensation remains vague. In the first part of this paper, our system resorts to experiments using isothermal titration calorimetry (ITC) to elucidate the energetic factors in protamine induced DNA condensation, supported by various microscopic and spectroscopic studies to gain structural insights. Although ITC is a powerful technique to study thermodynamic features of biomolecular interactions, no study has been reported on a protamine-DNA system so far.

In the second part, we have explored the effect of DNA binding drugs (DBDs) on DNA condensation. Although, substantial work has progressed in evaluating the drug-DNA interactions *in vitro*, it is often difficult to relate these results to pharmacological effects seen *in vivo* since the DNA is in packaged form inside cells. For example, condensed DNA may potentially block accessibility of drugs, hampering their biological activity. Therefore, how DNA condensed structure modifies drug’s binding, or how drug’s binding affects condensed structure is an important area of research. In recent studies it was observed that drugs can actually affect both chromatin structure and function^21,22^. Intercalating drug was shown to promote the release of histone H1 upon treatment with chromatin, disrupting the higher-order chromatin structure^23^. However, in order to find out if there is generic mechanism involved in the interference of DNA condensation by drugs^24^, more studies are required. A detailed biophysical characterization of the effect of DBD on DNA condensation will not only help to gain insights into the therapeutic value of drugs but also to improve our understanding about the mechanism that DNA adopts in condensation.

We have used netropsin (Net) and ethidium bromide (EtBr) as classical DNA binding drugs for their ability to bind DNA with dissimilar binding modes. Calf thymus DNA (^CT^DNA) containing AT/GC content (1.4:1) was appropriately chosen as biological DNA. We also used model sequences to gain further insights into sequence dependent effects. Net binds in the minor groove of DNA, preferably at A/T rich regions^25^, and is known to modulate DNA-protein interactions^26^. EtBr preferably intercalates in GC sequences^21^, however there are also reports indicating its intercalation in AT base pairs^27^. Also, it is known to interfere with topoisomerase activities by a DNA binding mechanism. Interestingly, in our studies, we found that Net and EtBr follow unique mechanism to interfere with the protamine-induced DNA condensation.

## Results

### Biophysical characterization of the protamine-DNA interaction

**Figure 1:**
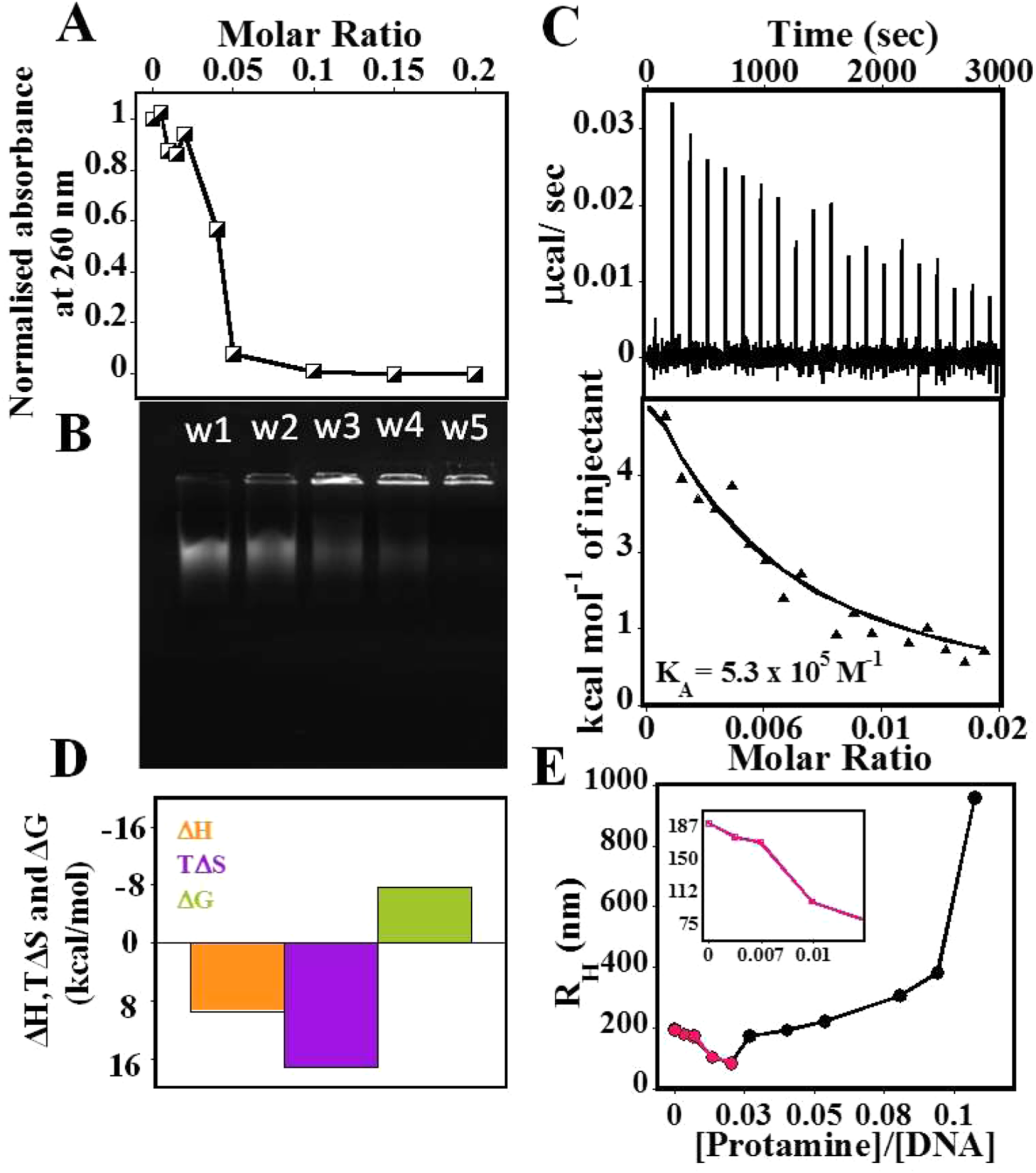
Characterization of the DNA condensation by protamine. (**A**) A Plot of a fraction of ^CT^DNA remaining in the solution as a function of increasing protamine concentrations (0–20 µM) measured by UV spectrophotometer. The concentration of ^CT^DNA was 50 µM/bp. (**B**) Gel electrophoresis showing the intensity band of ^CT^DNA in the absence (w1) and presence (w2–w5) of various protamine concentrations. (**C**) ITC thermogram showing the titration of ^CT^DNA (25 µM/bp) into protamine (300 µM), with endothermic heat showing condensation phase for ^CT^DNA. (**D**) Bar diagram showing the comparison of thermodynamic parameters. (**E**) DLS plot displaying the hydrodynamic radii vs protamine/DNA (P/D bp^−1^) molar ratio, showing the condensation and aggregation phases separately: inset gives the appearance of only condensation phase.

In UV-vis spectrophotometer, ^CT^DNA has a characteristic spectrum of the B-form DNA with a λ_max_ at 260 nm. In Fig. 1A, upon addition of increasing concentrations of protamine, there is a gradual decrease in the absorbance values at 260 nm, suggesting that DNA is undergoing condensation. Similar results have been reported earlier^3,28^. The plot levels off at ~0.05 protamine/DNA per base pair (P/D bp^−1^) molar ratio, indicating it to be the end point of DNA condensation. After addition of higher P/D bp^−1^, we could observe DNA precipitation. We also performed the gel electrophoresis experiments at various P/D bp^−1^ ratios (Fig. 1B). Here, the band intensity is directly proportional to the amount of ^CT^DNA remaining in the solution. Thus as shown in Fig. 1B, there is a gradual decrease in the band intensity of a free DNA (w1) with increasing protamine concentration from w2 to w5. To estimate the DNA fraction in each band, we considered the contrast of the free DNA (w1) to be 100% and plotted it against the molar ratio in Fig. S1. It was observed that the DNA band has disappeared beyond ~0.07 molar ratio (Fig. S1).

In ITC^29^, the binding isotherm obtained as a result of titration of ^CT^DNA into protamine (Fig. 1C) displayed endothermic heat, which touched zero baseline when all the available DNA molecules experienced the complete binding. The data were fitted to a single site model to obtain thermodynamic parameters (Fig. 1D) such as; binding constant (K_A_ =  5.5 × 10^5^ M^−1^), enthalpy (ΔH = 9.5 kcal/mol) and entropy (TΔS  = 17.3 kcal/mol/K). The enthalpy contributes unfavorably, whereas the entropy contributes favorably to the binding. The positive unfavorable enthalpy suggests the role of electrostatic interactions in protamine-induced DNA condensation^30^. Total entropy can have a negative contribution (conformational entropy) due to the collapse of the expanded DNA into condensed form^2^, and a positive contribution due to the release of counterions upon complexation (solvation entropy). However, overall positive entropy suggested that solvation entropy must be dominant over conformational entropy.

In order to study the changes in the average hydrodynamic radius of DNA as a result of the protamine binding, dynamic light scattering (DLS) experiments were performed. Here, the R_H_ data were obtained directly from the analyses of the auto-correlation function. The plot in Fig. 1E indicated the two stages in DNA condensation. In the first stage, from 0–0.05 P/D bp^−1^ molar ratio, (inset Fig. 1E) an average hydrodynamic radius (R_H_) of free DNA undergoes gradual decline (from 182.5 nm to 86 nm), indicating that DNA is undergoing condensation. Particle size for the compact DNA is in agreement with the condensation being monomolecular^12^. Beyond 0.05 ratio, initially, there is a very small increase in R_H_, which may corresponds to the ordering of the protamine-DNA molecules before they can form larger aggregates, as shown by an abrupt increase in the size of the protamine-DNA complex beyond 0.1 ratio of P/D. This biphasic nature indicates DNA undergoing intramolecular (monomolecular) to intermolecular (multimolecular) condensation^3^. At higher P/D, we observed DNA precipitation similar to UV results. Overall, the condensing ratio varies between 0.05–0.07, depending on the concentration of the DNA and the technique used.

**Figure 2:**
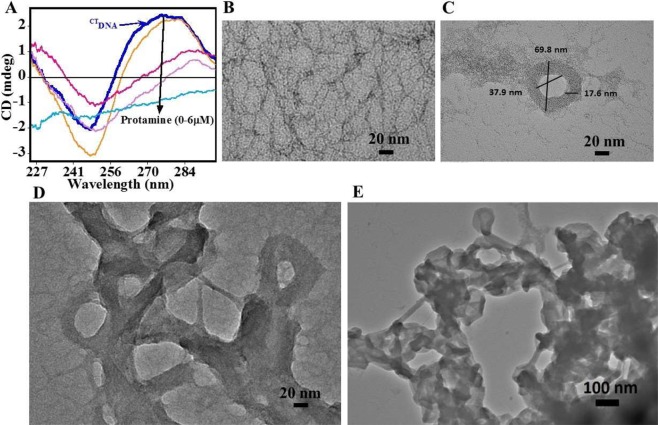
DNA Conformational studies. (**A**) Circular dichroism spectra of ^CT^DNA (50 µM/bp) in the presence of increasing concentration of protamine (0 µM–6 µM). (**B**–**E**) TEM images of protamine induced DNA condensates. (**B**) Elongated structures of free ^CT^DNA (140 µM/bp). (**C**) Monomolecular toroid as a result of protamine-DNA binding (^CT^DNA 140 µM/bp, protamine 2 µM). (**D**) Incomplete network film of DNA condensate. (**E**) Formation of multimolecular toroids containing subunits of toroidal size (^CT^DNA 140 µM/bp, protamine 11.2 µM).

DNA *in vivo* is generally present in condensed form, therefore, in order to understand these transitions here, we adopted CD spectroscopy and analyzed the changes in ^CT^DNA with and without protamine. The CD spectrum (Fig. 2A) of free ^CT^DNA showed a positive peak at 276 nm and a negative peak at 246 nm, a typical signature of the B-form of DNA^31^. On addition of protamine, a decrease in the intensity and red shift in the wavelength of CD spectra was observed. Such an alteration in the CD spectra corresponds to a conformational transition of B to ѱ-DNA, an essential condition for an ordered toroid-shaped DNA structure^12,32^. At higher protamine concentration, both the positive and negative bands were flattened confirming aggregation of DNA.

TEM with its spatial resolution helps to visualize and measure the higher ordered packaging of DNA. The molecules of ^CT^DNA without protamine (Fig. 2B) appear in the form of elongated coil shape^8^. However, the addition of protamine to DNA (Fig. 2C) that induced strong variation in CD, resulted into collapsed (compact) structure with an average outer diameter of 80 nm and the inner diameter of 38 nm, a characteristic of the toroidal form of DNA^4^. Here, we also observed another type of species with thin and incomplete network film, representing the kinetically trapped incomplete condensed states of DNA (Fig. 2D). However, at higher P/D bp^−1^ ratio (~0.12), the morphology appears to have much greater dimensions, a characteristic of intermolecular aggregates (Fig. 2E). The average diameter of subunits in intermolecular aggregates is similar to the outer diameter of the monomolecular toroids. Overall, the results suggested that the DNA can form toroidal forms at lower protamine concentration, and large sized ordered aggregates at higher protamine concentration.

### Interaction of protamine with the drug-bound DNA

**Figure 3:**
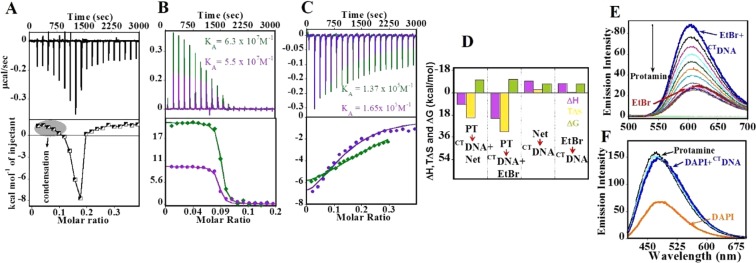
ITC Binding studies of ^CT^DNA. The titration of protamine (200 µM) into (**A**) ^CT^DNA (100 µM/bp), (**B**) protamine (PT) (80 µM) into () ^CT^DNA (100 µM/bp) + Net (60 µM) and () ^CT^DNA (100 µM/bp) + EtBr (60 µM). (**C**) Titration of () Net (200 µM) and () EtBr (150 µM) into ^CT^DNA (100 µM/bp). The top panel represents the raw data for the sequential injection of ligands into sample cell and the bottom panel shows the integrated heat data after correction of heat of dilution. (**D**) Bar diagram with the comparison of thermodynamic parameters in the binding events. (**E**,**F**) Fluorescence displacement assay. Fluorescence intensity changes accompanying titration of protamine into (**E**) EtBr bound ^CT^DNA and (**F**) DAPI bound ^CT^DNA. The concentration of ^CT^DNA, EtBr and DAPI was 50 µM each.

In order to understand the thermodynamic basis of the effect of DBD on DNA condensation, the drug- bound and naked ^CT^DNA was titrated with protamine in ITC (Fig. 3). The binding isotherm obtained (Fig. 3A) by titrating protamine into naked ^CT^DNA was biphasic, displaying the initial five endothermic peaks, followed by four exothermic peaks, before finally returning to zero baseline as a result of complete saturation of binding sites. The ITC titration here is reversed compared to the titration discussed in Fig. 1C. The data could not be fitted to any model due to the complexity of the binding curve, which involved the condensation (endothermic) as well as aggregation (exothermic) steps. The titration of protamine into the Net-bound ^CT^DNA (Fig. 3B) resulted into simple monophasic sigmoidal curve, with an overall binding driven by endothermic heat (ΔH = 9.6 kcal/mol) and the positive entropy (TΔS = 20.1 kcal/mol; Table 1)^30^. In the case of the EtBr-bound ^CT^DNA (Fig. 3B), the protamine binding resulted into higher unfavorable ΔH (20.9 kcal/mol) and favorable TΔS (31.58 kcal/mol). The strong positive entropy terms in both the cases suggested electrostatic interactions to be dominant^30^. It also suggests that solvation entropy is dominant over conformational entropy. Although there was a significant difference in the values of enthalpy and entropy for Net-bound ^CT^DNA and EtBr-bound ^CT^DNA, enthalpy-entropy compensation resulted in their similar ΔG values (Table 1). This was also confirmed by K_A_ values, which are comparable for EtBr-bound-DNA (6.36 × 10^7^ M^−1^) and Net-bound-DNA (5.54 × 10^7^ M^−1^).

**Table 1:** Thermodynamics parameters for ^CT^DNA.

Systems	N	K (M^−1^)	ΔH (kcal/mol)	TΔS (kcal/mol)	ΔG (kcal/mol)
Protamine + (EtBr-^CT^DNA)	0.08 ± 0.0003	6.36 × 10^7^	20.9 ± 0.17	31.5	−10.6
EtBr + ^CT^DNA	0.26 ± 0.002	1.37 × 10^5^	−7.4 ± 0	−0.41	−6.9
Protamine + (Net-^CT^DNA)	0.078 ± 0.006	5.54 × 10^7^	9.6 ± 0.14	20.1	−10.5
Net + ^CT^DNA	0.14 ± 0.005	1.65 × 10^5^	−9.4 ± 0	−2.3	−7.1

Experiments were carried out in Hepes buffer at 25 °C. Errors for ΔH and N are fitting errors from ITC. Errors for K, ∆G, and T∆S are 15–20%.

In order to understand the precise molecular level effect of DBD on DNA condensation, we also performed separate binding studies of Net and EtBr with the ^CT^DNA (Fig. 3C). Thermodynamic parameters for the same are presented in Fig. 3D with a complete list in Table 1. Net displayed slightly higher binding affinity (K_A_ = 1.64 × 10^5^ M^−1^) for ^CT^DNA compared to EtBr (K_A_ = 1.37 × 10^5^ M^−1^).

Fluorescence displacement assay (FDA) was performed by titrating protamine into the drug-bound ^CT^DNA to test the competition between a drug and protamine for the DNA binding sites. In Fig. 3E, free EtBr gives positive intensity upon excitation at 480 nm. The binding of EtBr with ^CT^DNA resulted in a significant increase in the intensity compared to its free species. However, the gradual addition of protamine to EtBr-bound ^CT^DNA resulted in the reduction in the intensity, suggesting a displacement of EtBr from the intercalation site. Since Net is not fluorescent, we replaced it with another well-known minor groove binder, DAPI, which has very good quantum yield as well as similar DNA binding affinity as Net^29^. A test experiment in Fig. S4 also shows that DAPI can bind to protamine-DNA complex equally well. In Fig. 3F, the fluorescence intensity of ^CT^DNA-bound DAPI was greater than only DAPI, suggesting that DAPI bound strongly to ^CT^DNA. When protamine was titrated into DAPI-bound ^CT^DNA, no substantial alteration in the fluorescence intensity was observed, which indicates that protamine failed to displace DAPI from the minor groove.

### Interaction of drugs with the protamine-bound DNA

**Figure 4:**
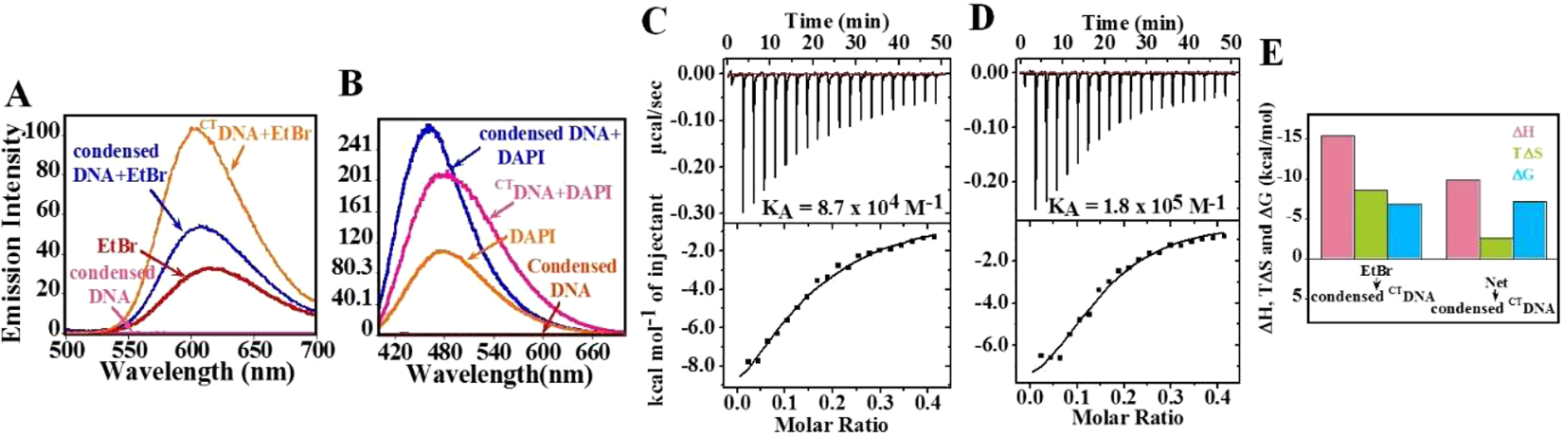
Reverse fluorescence displacement assay. Fluorescence intensity changes accompanying titration of (**A**) EtBr (50 µM) to condensed DNA. (**B**) DAPI (50 µM) to condensed DNA. (**C**–**E**) Reverse ITC binding studies. Representative raw ITC and integrated heat data for binding of (**C**) EtBr (200 µM) (Stoichiometry, N = 0.13). (**D**) Net (200 µM) (Stoichiometry, N = 0.15) with condensed DNA (^CT^DNA + protamine) respectively. The top panel corresponds to raw data and the bottom panel shows the integrated heat for each injection with respect to the molar ratio of total ligand to the total ^CT^DNA and (**E**) Bar diagram describing the variation of magnitude of thermodynamic parameters in the binding event.

Next, we performed reverse fluorescence displacement assay by titrating DBD into the condensed form of DNA (P/D bp^−1^ complex at 0.1 ratio) to test if a drug can access its binding sites in the condensed DNA. As shown in Fig. 4A, the fluorescence intensity resulted from the binding of EtBr to condensed DNA is greater than free EtBr, which clearly indicates that EtBr is able to access intercalation sites in condensed DNA. However, the resultant intensity is smaller than the ^CT^DNA-EtBr complex, suggesting that not all of the intercalating sites are accessible to EtBr in the condensed DNA. On the contrary, DAPI was able to achieve very strong fluorescence intensity on binding with condensed DNA, almost similar to its binding with naked DNA. This indicates that DAPI can access all the minor groove sites in condensed DNA (Fig. 4B) just as naked DNA.

Further, in order to obtain the thermodynamic parameters associated with the binding of a drug with condensed DNA, we performed reversed ITC experiments (Fig. 4C–E). It was observed that K_A_ for the binding of the Net with condensed DNA (1.8 × 10^5^ M^−1^) is comparable to its K_A_ for naked ^CT^DNA (1.65 × 10^5^ M^−1^). On the contrary, K_A_ for the binding of EtBr for condensed DNA (8.7 × 10^4^ M^−1^) is slightly lower than its K_A_ with naked ^CT^DNA (1.37 × 10^5^ M^−1^). The binding here is enthalpically favorable and entropically unfavorable (Fig. 4E).

### Effect of drugs on the interaction between protamine and AT/GC rich DNA sequences

**Figure 5:**
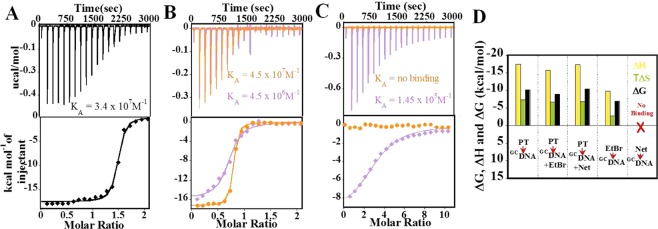
ITC Binding studies of ^GC^DNA. The titration of protamine (100 µM) into (**A**) ^GC^DNA (10 µM), (**B**) () ^GC^DNA (10 µM) + EtBr (20 µM) and () ^GC^DNA (10 µM) + Net (20 µM), (**C**) The titration of () Net (200 µM) and () EtBr (500 µM) into ^GC^DNA (10 µM). The top panel represents the raw data for the sequential injection of ligands into sample cell and the bottom panel shows the integrated heat data after correction of heat of dilution. (**D**) Bar diagram with the comparison of thermodynamic parameters in the binding events.

In order to understand the precise role of DNA sequences in condensation, we used two duplex sequences (^GC^DNA and ^AT^DNA, ‘Materials’ section) in our study. ITC measurements (Fig. 5A) displayed a very strong binding affinity between protamine and ^GC^DNA (K_A_ = 3.4 × 10^7^; M^−1^, ΔH = −17.5 kcal/mol; TΔS = −7.3 kcal/mol) dominated by favorable enthalpy. In Fig. 5B, protamine showed 7–8 times weaker affinity with EtBr-bound ^GC^DNA (4.5 × 10^6^ M^−1^, ΔH = −15.7 kcal/mol, TΔS −6.7 kcal/mol), also driven by favorable enthalpy. Interestingly, with Net-bound ^GC^DNA (Fig. 5B), protamine resulted in similar binding parameters (K_A_ = 4.5 × 10^7^ M^−1^; ΔH = −17.3 kcal/mol; TΔS = −6.9 kcal/mol) as with naked ^GC^DNA. The binding of Net with ^GC^DNA was found to be negligible, showing only background signal that was associated with the heats of dilution when the Net was titrated into a buffer (Fig. 5C). On the other hand, EtBr bound fairly strongly to ^GC^DNA (Fig. 5C). Complete thermodynamic parameters for the binding are compared in Fig. 5D and Table S1.

**Figure 6:**
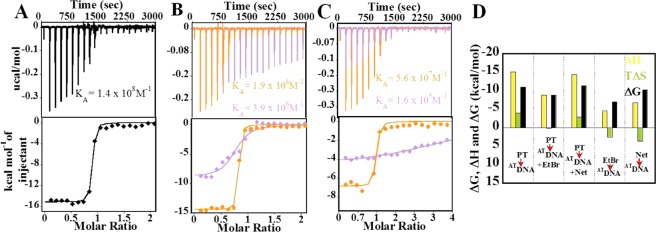
ITC Binding studies of ^AT^DNA. The titration of protamine (100 µM) into (**A**) ^AT^DNA (10 µM), (**B**) () ^AT^DNA (10 µM) + EtBr (20 µM) and () ^AT^DNA (10 µM) + Net (20 µM), (**C**) The titration of () Net (200 µM) and () EtBr (200 µM) into ^AT^DNA (10 µM). The top panel represents the raw data for the sequential injection of ligands into sample cell and the bottom panel shows the integrated heat data after correction of heat of dilution. (**D**) Bar diagram with the comparison of thermodynamic parameters in the binding events.

The binding of protamine to ^AT^DNA (Fig. 6A) was enthalpically driven (ΔH = −15.1 kcal/mol) and entropically opposed (TΔS = −4.0 kcal/mol), resulting into strong binding affinity (K_A_ = 1.4 × 10^8^ M^−1^). The protamine binding to Net-bound ^AT^DNA (Fig. 6B) resulted (K_A_ = 1.89 × 10^8^ M^−1^; ΔH = −14.4 kcal/mol; TΔS = −2.9 kcal/mol) in almost similar parameters as with naked ^AT^DNA, however, significantly stronger than to EtBr-bound ^AT^DNA (Fig. 6B; K_A_ = 3.9 × 10^6^ M^−1^; ΔH = −8.8 kcal/mol; TΔS = 0.1 kcal/mol) (Table S2). This suggests that the strong association of Net with ^AT^DNA did not distort the protamine binding to ^AT^DNA thermodynamically. In Figs 5C and 6C, the binding constants obtained for EtBr with the ^GC^DNA and ^AT^DNA were almost similar (K_A_ = 1.45 × 10^5^ M^−1^ and K_A_ = 1.65 × 10^5^ M^−1^ respectively; Tables S1 and S2), in agreement with the previous reports^33^.

### Effect of drugs on the structure of DNA condensates

**Figure 7:**
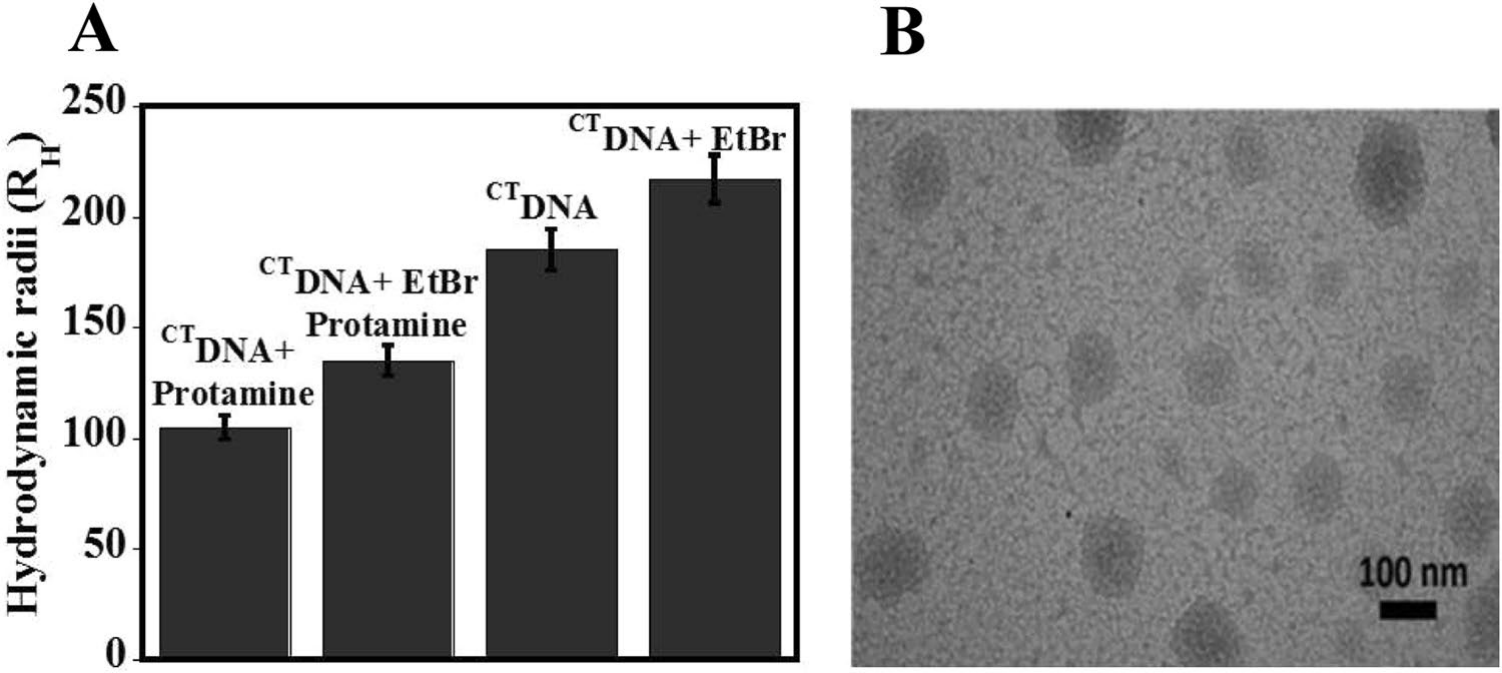
Effect of EtBr on DNA condensates. (**A**) Comparison of hydrodynamic radius of only ^CT^DNA; Complex of ^CT^DNA and protamine; complex of ^CT^DNA and EtBr; and ternary complex of ^CT^DNA, EtBr and protamine obtained from light scattering measurements. The concentration of ^CT^DNA was 138 µM and protamine 2 µM. (**B**) TEM image of DNA (140 µM) in the presence of protamine (11.2 µM) and EtBr (70 µM).

Apart from thermodynamic insights, DLS and TEM experiments were performed to identify the effect of a drug on structural changes to DNA condensates. Figure 7A shows the comparison of R_H_ values for various states of DNA using DLS. Here, the complex of protamine- ^CT^DNA (R_H_ = 105 nm) has the most compact state, whereas ^CT^DNA-EtBr (R_H_ = 217.5) has the most stretched out state. However, in the case of ternary complex (^CT^DNA-EtBr-protamine), the size (R_H_ = 135 nm) was found to be in between these two states. In TEM, (Fig. 7B) EtBr alters the conformation of a toroid with the loss of inner diameter from the structure, resulting into somewhat disordered conformation. The overall size (R_H_ = 89–135 nm) of these modified toroids was found to be higher than typical size found in Fig. 2C.

## Discussion

Generally, the DNA condensation is difficult to distinguish rigorously from aggregation or precipitation^2^. While condensation of single molecules is possible, it is also very common to observe several molecules that are incorporated into the condensed structure^2–4^. In the presence of protamine in TEM (Fig. 2), we observed single DNA molecules being collapsed into toroids, as well as multiple molecules being arranged into orderly aggregates having a finite size. The aggregated form of DNA consists of toroidal condensates networked through DNA strands (Fig. 2E). CD results (Fig. 2A) suggested conformational transition in DNA (B to ѱ) to be responsible for tightly packaged self-assembly of toroids as also exemplified by TEM. The ^CT^DNA contains a substantial population of GC stretches, which can act as nucleation centers to promote ѱ- form as has been reported earlier^34^. Further, B to ѱ junctions increase the bending properties of the DNA^34,35^, which have a strong tendency to promote condensation.

ITC binding results (Fig. 1D) indicated the role of counterion condensation mechanism^2^ in protamine-DNA interaction. Also, the positive values for enthalpy as well as entropy (Fig. 1D) suggested the binding of protamine to be accompanied by an initial disruption of the structured water layer around the DNA backbone. The resultant complex is then surrounded by a newly formed solvent cage, which lacks the polarity of the hydration shell of naked DNA. If Parsegian and co-workers^36^ model is followed, protamine will cause the DNA-DNA attraction by inciting the rearrangement of surface water by forming regions of hydration attraction between DNA molecules until the whole structure collapses into condensed particles. This signifies the role of hydration force in condensation^22^.

In the absence of X-ray structure of the protamine-DNA complex, there are confusing views about what kind of binding mode as well as the structure protamine adapts in bound form. The random coil structure of protamine in CD (data not presented here) and endothermic heat in ITC (Fig. 1C), together suggested that protamine binds externally to DNA backbone in an extended conformation. Many reviews have established that there is clear preference for pairing between Arg and guanine^37^. Arg contains the guanidinium moiety comprising three amine groups (donors), which have potential to form bidendate hydrogen bonding with the phosphate oxygens, and with guanine N7 and O6 (acceptors) in the major groove, resulting into stronger binding^37,38^. Similar binding of Ni (II) with guanine in the major groove has resulted in DNA condensation through B- to ѱ-DNA transformation^39^. Several other Arg rich peptides have also been shown to interact with DNA through such interactions^40^. Furthermore, occupancy of arginines in the major groove would also help to reduce the overall volume required for condensed DNA inside cells^41^. Thus, apart from non-specific external binding, protamine can also interact specifically with the DNA bases in the major groove through Arg residues.

Combining our experimental and literature data, the mechanism of protamine-induced DNA condensation can be concluded as follows. Protamine binds externally to DNA by electrostatic forces. The average binding ratio of ~0.05 (protamine/DNA bp^−1^) obtained from Fig. 1 (UV-vis, DLS and Gel studies) implies that one protamine molecule binds to approximately 20 base pairs of DNA (1/20 = 0.05). It is possible that, when first guanidium group (from Arg) interacts with the DNA on one side, due to steric constraints, the next adjacent guanidium group is forced to interact with the DNA on the other side^42^. Thus it is reasonable to consider that a protamine binds two DNAs, one on each side (each DNA with ~10 base pairs) so that the adjacent DNAs share the protamines. Successively, guanidinium groups on one side of protamine will form hydrogen bonds with O6 and N7 atoms in guanine residues in the major groove. At much higher protamine concentration arginines can favour the interaction of multiple DNA molecules, thereby allowing the formation of multimolecular aggregates. The external mode of protamine also helps to bridge molecules. Here, DNA slowly undergoes aggregation and precipitation under relatively high concentrations.

The interactions between drugs and condensed DNA may help in predicting the potential therapeutic significances of such interactions. Using various biophysical experiments, we compared the effect of drugs on naked and condensed DNA. The binding of protamine with naked ^CT^DNA displayed a complex isotherm involving condensation as well as aggregation (Fig. 3A). In contrast, with the Net-bound ^CT^DNA, a distinct monophasic curve was obtained (Fig. 3B), which has essentially ruled out aggregation phase. The result implies that the protamine- ^CT^DNA binding was modified successfully by Net. Interestingly, in the FDA (Fig. 3F), protamine could not displace DAPI from the (AT) minor groove, suggesting that the minor groove binding agent and protamine must have independent binding sites on ^CT^DNA. The similar stoichiometry for protamine-^AT^DNA (N = 0.8) and protamine-^AT^DNA-Net (N = 0.7) complexes (Table S2) endorses this view. This also helped to rule out an earlier hypothesis that Arg residues of protamine bind in the minor groove of DNA^41^. Because, if Arg residues were to bind in the minor groove, they would have prohibited DAPI/Net from occupying the minor groove.

However, the question remains, how protamine- ^CT^DNA binding (Fig. 3A) was altered by the presence of minor groove binding agent? First, Net stabilizes DNA through van der Waals interactions as well as through H-bond interaction between NH groups of the amidine and N_3_ of adenine and O_2_ of thymine in the minor groove^25^. Net is penetrated deeply in the minor groove and leaves less room for conformational fluctuations. Thus, it is capable of inducing pronounced stiffness in the DNA through improved DNA stability (Fig. S2), which eventually help in modifying the extent of condensation by protamine. Secondly, Net/DAPI is not competing directly with protamine, which means it may be influencing the protamine binding through some other mechanism, such as an allosteric modulation. Drug interference of the protein-DNA interaction by an allosteric modulation has been well reported in the literature^43,44^. Diamidines have also been shown to modulate the binding of the major groove binding transcription factor in similar fashion^45^. Moreover, it has been revealed through electrophoretic mobility studies that Net can exert DNA structural effects such as the bending of the double helix in the minor groove^46,47^. Based on these observations, Net most likely bends DNA in the minor groove^48^, opposing the degree of protamine-induced DNA bending in the major groove. This conflicting effect may reduce the flexibility of the DNA, due to which there could be subdued protamine crosslinking and aggregation. Kinetic studies in Fig. S3 also supported the suppression of multimolecular aggregates in the presence of a drug.

In reverse titration experiments, the binding of Net with condensed DNA and naked DNA was found to be similar (Figs 3C and 4D), suggesting that i) Net has full access to its binding sites in condensed DNA ii) protamine interactions are restricted to phosphate backbone and major groove sites only, and iii) during DNA packaging, minor groove sites are well exposed to the solvent. A previous report on the crystal structure of ѱ- DNA, which has revealed that the narrow minor groove character persists during B to ѱ- DNA transformation^49,50^, supports these observations. The ability to access DNA sites with equal probability in naked as well as condensed DNA could be the generic feature of this class of compounds. Thus, in cellular context, groove binder may inflict better inhibitory effect on DNA-binding proteins and hence better gene regulation.

Protamine was fully able to displace EtBr from the complex (Fig. 3E), indicating that protamine and EtBr share common binding sites on DNA. This is also well supported by the Tm studies in Fig. S2. The binding thermodynamics of protamine with ^CT^DNA, ^GC^DNA, as well as ^AT^DNA was also tailored significantly in the presence of EtBr (Figs 3–6, S2). The large positive entropy (protamine-EtBr-bound ^CT^DNA; Fig. 3D) suggests that the ternary complex is more flexible, perhaps because protamine is less able to form cross-links with DNA in the presence of EtBr, preventing the ordered multimolecular condensation. This is also supported by more loosened, partially unfolded toroidal structures observed for this complex in TEM (Fig. 7). EtBr provokes these changes due of its ability to intercalate between GC as well as AT base-pairs^33,51^ and induce DNA conformational changes. Also, its ability to block the guanine binding sites of protamine on the DNA, indicated by the lower stoichiometry of the protamine-^GC^DNA-EtBr complex (~0.7) compared to protamine-^GC^DNA (~1.5) complex (Table S1), can prevent B to Ψ transition. Thus, the ability of protamine to force DNA condensation experiences resistance from EtBr.

Also, EtBr was shown to bind weakly to the condensed DNA (Fig. 4A,C) probably due to two reasons. First, separation of a base pair for intercalation in condensed DNA would be thermodynamically more unfavorable. This is indicated by the higher negative entropy for the binding of EtBr with condensed DNA (TΔS = −8.6 kcal/mol) compared to naked DNA (TΔS = −0.41 kcal/mol) (Fig. 3D and 4E). Secondly, due to significant distortion of GC bases^49^ in condensed DNA, EtBr can probably access AT base pairs for intercalation, resulting in an inadequate increase in the fluorescence intensity (Fig. 4A). Further, based on direct and reverse experiments, it can be concluded that EtBr pre-bound with the DNA was more effective in inhibiting DNA condensation than when it was added to the already condensed DNA^52^. In the previous reports, intercalators, which were found to interfere with the stability of nucleoprotein particles, may have induced similar alterations in the DNA^23^.

In summary, with the help of energetic and structural factors, we proposed a DNA condensation mechanism, together with one of the most important findings that intercalator and minor groove binder adapt different mechanism to influence the DNA packaging. An understanding of the types of chemical interactions between drugs and packaged DNA may help in predicting the potential therapeutic or physiological consequences of such interactions. Moreover, such knowledge should be a part of future drug-design ideas.

## Materials and Methods

Calf thymus DNA, protamine sulfate salt from salmon, Ethidium bromide, netropsin dihydrochloride and 4′, 6-diamidino-2-phenylindole dihydrochloride (DAPI) were purchased from Sigma Aldrich Co. and used without further purification. Self-complementary primers for ^GC^DNA i.e. 5′-GCGCGCGCGC-3′ and 5′-GCGCGCGCGC-3′ and for ^AT^DNA i.e. 5′-GCAAATTTGC-3′ and 5′-GCAAATTTGC-3′ were purchased from Integrated DNA technologies. Their duplexes were prepared by heating the two complementary strands at 85–90 °C, for 5 min, and then slowly cooling to room temperature. The final concentration of the DNA was determined spectrophotometrically by measuring absorbance at 260 nm using a molar extinction coefficient of ε_260_ = 13200 bpM^−1^ cm^−1^ for ^CT^DNA^29^, ε_260_ = 158840.8 M^−1^ cm^−1^ for ^GC^DNA and ɛ_260_ = 153441.4 M^−1^ cm^−1^ per strand for ^AT^DNA. The stock solution of 1 mM each of DAPI, Net, EtBr and protamine were prepared in HPLC water.

### Sample preparation

Protamine has total 32 amino acids, containing 21 Arg residues (giving it 21 positive charges). We have expressed the concentration ^CT^DNA in base pairs. For a single base pair of DNA, two negative charges are present (due to phosphates). Different binding molar ratios of protamine/DNA bp^−1^ were used throughout the paper. For examples; the binding molar ratio of 0.05 (protamine/DNA bp^−1^) implies that 1 protamine molecule binds with 20 bp of DNA (1/20 = 0.05). For shorter duplex DNAs, the concentration was expressed as strand concentration.

### UV Spectroscopy measurements

The UV measurements were performed in Cary 100 UV-Vis spectrophotometer by titrating protamine into the fixed DNA concentration. Each sample was prepared using 10 mM Hepes buffer, 100 mM NaCl at pH 7.4. After incubation (at 4 °C for 2 hours), the aliquots were centrifuged at 60000 rpm for 10 min. The amount of DNA condensation was monitored by monitoring the change in absorbance value at 260 nm, which was then normalized and plotted as a function of the molar ratio of protamine/DNA. Melting studies were done over a temperature range of 25 °C–90 °C at a constant concentration of 50 µM ^CT^DNA complexed with 3 µM of protamine in the presence and absence of EtBr or Net. In kinetic measurements, the effect of drug was monitored on DNA condensates (0.1 protamine/DNA molar ratio) as function of time.

### ITC

ITC experiments were carried out on MicroCal iTC_200_ system (Malvern Instruments Ltd., UK) at 25 °C temperature, the details of which are published previously^53,54^. In case of direct titration, protamine was titrated into the drug-bound ^CT^DNA, and in reverse titration, a drug was titrated into the protamine-^CT^DNA complex. In case of shorter DNA sequence, protamine (100 µM) was titrated into DNA (10 µM) in the absence and presence of drug (20 µM). Control experiments were performed by titrating protamine or drug into a buffer to ignore the contribution of buffer interaction. The results were analyzed using Origin software and fitted to a one set of site model to give stoichiometry (N), binding constant (K_A_) and enthalpy change (ΔH). Using K_A_ and ΔH, the Gibbs free energy change (ΔG = −RT ln K_A_) and entropy change (ΔG = ΔH − TΔS) can be calculated.

### DLS

DLS measurements were performed on LS Spectrometer by LS instruments, employing a 21 mV He-Ne Laser operating at a wavelength of 632.8 nm, at a scattering angle of 90° with two highly sensitive avalanche photodiode (APD) detectors at 25 °C. The instrument was placed on a vibration isolation table. The distribution of apparent radius R_H_ were obtained from the distribution of mean apparent translational diffusion coefficients (D_T_) via

$${{\rm{R}}}_{{\rm{h}}}={\rm{kT}}/(6{{\rm{\pi }}{\rm{\eta }}{\rm{D}}}_{{\rm{T}}})/2$$

where k is the Boltzmann constant, η is the solvent viscosity which is assumed to be that of water and T is the temperature. To compare the effect of EtBr on ^CT^DNA –protamine complexation, hydrodynamic radii (R_H_) of only ^CT^DNA, protamine-^CT^DNA complex, DNA - protamine-EtBr ternary complex were measured. Concentrations of ^CT^DNA, protamine and EtBr were kept constant in all the samples. For the preparation of DNA-EtBr-protamine ternary complex, 50 µM EtBr was mixed with 138.2 µM of ^CT^DNA. After waiting for 5 mins, 2 µM protamine was added to it and measurement was recorded.

### Gel Electrophoresis

The samples of ^CT^DNA were prepared in 10 mM Hepes buffer, 100 mM NaCl by mixing with different concentration of protamine (0–10 µM). 1.5% agarose gel was prepared to contain 0.5 µg/mL of EtBr for DNA staining during the run. Final condensing stage of ^CT^DNA was measured by the relative density of each DNA band, which is proportional to the DNA concentration in the band using ImageJ software.

### CD

Circular dichroism experiments were carried using Chirascan Applied Photophysics spectropolarimeter. All the experiments were carried at room temperature with quartz cuvette of 3 mm path length. ^CT^DNA at a fixed concentration of 50 µM was titrated with the varied concentrations of protamine (0–6 µM) at pH 7.4, 10 mM Hepes buffer with 100 mM NaF. The measurements were taken at a wavelength range of 220 nm–300 nm and at a scan speed of 75 nm/min. 5 scans were recorded and computer averaged. Baseline spectra of a buffer was always subtracted from the spectra. The measurement was carried out by incubating the samples at 4 °C for 2 hours.

### TEM

TEM micrographs were recorded using a transmission electron microscope (JEM-2100F JEOL) at an operating voltage of 200 kV. Samples prepared in 10 mM Hepes buffer were drop cast on carbon-coated copper grid with 300 mesh size and dried. It was stained with uranyl acetate and dried again. The grid was placed in the sample compartment to record the micrographs.

### FDA

FDA was performed on Cary eclipse fluorescence spectrophotometer at a scanning speed of 600 nm/min. In direct displacement fluorescence assay, the protamine was titrated into a mixture of ^CT^DNA and EtBr by exciting EtBr at 480 nm. Similar experiments were performed using DAPI as a probe by using excitation wavelength of 375 nm. In Reverse displacement assay, drug was titrated into already condensed DNA (protamine/DNA molar ratio of 0.1).

## Supplementary information

Supplementary information

## References

[1] Gosule LC, Schellman JA (1976). Compact form of DNA induced by spermidine. Nature.

[2] Bloomfield, V. A. DNA condensation by multivalent cations. *Biopolymers* **44**, 269–282, doi:10.1002/(SICI)1097-0282(1997)44:3<269::AID-BIP6>3.0.CO;2-T (1997). 10.1002/(SICI)1097-0282(1997)44:3<269::AID-BIP6>3.0.CO;2-T 9591479

[3] Katz AM (2017). Spermine Condenses DNA, but Not RNA Duplexes. Biophys J.

[4] Lin Z (1998). The observation of the local ordering characteristics of spermidine-condensed DNA: atomic force microscopy and polarizing microscopy studies. Nucleic Acids Res.

[5] DeRouchey J, Parsegian VA, Rau DC (2010). Cation charge dependence of the forces driving DNA assembly. Biophys J.

[6] Matulis D, Rouzina I, Bloomfield VA (2002). Thermodynamics of cationic lipid binding to DNA and DNA condensation: roles of electrostatics and hydrophobicity. J Am Chem Soc.

[7] Smith RJ, Beck RW, Prevette LE (2015). Impact of molecular weight and degree of conjugation on the thermodynamics of DNA complexation and stability of polyethylenimine-graft-poly(ethylene glycol) copolymers. Biophys Chem.

[8] Fan Y (2017). DNA Condensation Induced by a Star-Shaped Hexameric Cationic Surfactant. ACS Appl Mater Interfaces.

[9] Upadhyay, S. K. Binding and thermodynamics of REV peptide-ctDNA interaction. *Biopolymers* **108**, 10.1002/bip.22902 (2017). 10.1002/bip.22902 27353011

[10] Chen D (2005). Condensed mitotic chromatin is accessible to transcription factors and chromatin structural proteins. J Cell Biol.

[11] Mintzer MA, Simanek EE (2009). Nonviral vectors for gene delivery. Chem Rev.

[12] Li C (2013). Effective and reversible DNA condensation induced by a simple cyclic/rigid polyamine containing carbonyl moiety. J Phys Chem B.

[13] Korolev N, Berezhnoy NV, Eom KD, Tam JP, Nordenskiöld L (2009). A universal description for the experimental behavior of salt-(in)dependent oligocation-induced DNA condensation. Nucleic Acids Res.

[14] Nayak AK, Mishra A, Jena BS, Mishra BK, Subudhi U (2016). Lanthanum induced B-to-Z transition in self-assembled Y-shaped branched DNA structure. Sci Rep.

[15] Rodman TC, Pruslin FH, Allfrey VG (1984). Protamine-DNA association in mammalian spermatozoa. Exp Cell Res.

[16] DeRouchey JE, Rau DC (2011). Role of amino acid insertions on intermolecular forces between arginine peptide condensed DNA helices: implications for protamine-DNA packaging in sperm. J Biol Chem.

[17] DeRouchey J, Hoover B, Rau DC (2013). A comparison of DNA compaction by arginine and lysine peptides: a physical basis for arginine rich protamines. Biochemistry.

[18] Hud NV, Milanovich FP, Balhorn R (1994). Evidence of novel secondary structure in DNA-bound protamine is revealed by Raman spectroscopy. Biochemistry.

[19] Brewer LR, Corzett M, Balhorn R (1999). Protamine-induced condensation and decondensation of the same DNA molecule. Science.

[20] DeRouchey JE, Rau DC (2011). Salt effects on condensed protamine-DNA assemblies: anion binding and weakening of attraction. J Phys Chem B.

[21] Banerjee A (2014). The DNA intercalators ethidium bromide and propidium iodide also bind to core histones. FEBS Open Bio.

[22] Majumder P, Dasgupta D (2011). Effect of DNA groove binder distamycin A upon chromatin structure. PLoS One.

[23] McMurray CT, van Holde KE (1986). Binding of ethidium bromide causes dissociation of the nucleosome core particle. Proc Natl Acad Sci USA.

[24] Reich Z, Ghirlando R, Arad T, Weinberger S, Minsky A (1990). Extensive interference of DNA packaging processes affected by chemotherapeutic drugs. J Biol Chem.

[25] Nunn CM, Garman E, Neidle S (1997). Crystal structure of the DNA decamer d(CGCAATTGCG) complexed with the minor groove binding drug netropsin. Biochemistry.

[26] Miao Y, Cui T, Leng F, Wilson WD (2008). Inhibition of high-mobility-group A2 protein binding to DNA by netropsin: a biosensor-surface plasmon resonance assay. Anal Biochem.

[27] Mishra A, Ekka MK, Maiti S (2016). Influence of Ionic Liquids on Thermodynamics of Small Molecule-DNA Interaction: The Binding of Ethidium Bromide to Calf Thymus DNA. J Phys Chem B.

[28] Yu H, Ren J, Qu X (2007). Time-dependent DNA condensation induced by amyloid beta-peptide. Biophys J.

[29] Freyer MW, Buscaglia R, Nguyen B, Wilson WD, Lewis EA (2006). Binding of netropsin and 4,6-diamidino-2-phenylindole to an A2T2 DNA hairpin: a comparison of biophysical techniques. Anal Biochem.

[30] Matulis D, Rouzina I, Bloomfield VA (2000). Thermodynamics of DNA binding and condensation: isothermal titration calorimetry and electrostatic mechanism. J Mol Biol.

[31] Kypr J, Kejnovská I, Renciuk D, Vorlícková M (2009). Circular dichroism and conformational polymorphism of DNA. Nucleic Acids Res.

[32] Dey D, Maiti C, Maiti S, Dhara D (2015). Interaction between calf thymus DNA and cationic bottle-brush copolymers: equilibrium and stopped-flow kinetic studies. Phys Chem Chem Phys.

[33] Ruel, M. E. *Insights into the Relative DNA Binding Affinity and Preffered Binding Mode of Homologous Compounds Using Isothermal Titration Calorimetry (ITC)*. 129–152 (INTECH Open Access Publisher, 2013).

[34] Ma C, Sun L, Bloomfield VA (1995). Condensation of plasmids enhanced by Z-DNA conformation of d(CG)n inserts. Biochemistry.

[35] Sitko JC, Mateescu EM, Hansma HG (2003). Sequence-dependent DNA condensation and the electrostatic zipper. Biophys J.

[36] Parsegian VA, Rand RP, Rau DC (2000). Osmotic stress, crowding, preferential hydration, and binding: A comparison of perspectives. Proc Natl Acad Sci USA.

[37] Luscombe NM, Laskowski RA, Thornton JM (2001). Amino acid-base interactions: a three-dimensional analysis of protein-DNA interactions at an atomic level. Nucleic Acids Res.

[38] Kono H, Sarai A (1999). Structure-based prediction of DNA target sites by regulatory proteins. Proteins.

[39] Abrescia NG (1999). Structure of the oligonucleotide d(CGTATATACG) as a site-specific complex with nickel ions. Nucleic Acids Res.

[40] Mann A (2011). Differences in DNA condensation and release by lysine and arginine homopeptides govern their DNA delivery efficiencies. Mol Pharm.

[41] Balhorn R (1982). A model for the structure of chromatin in mammalian sperm. J Cell Biol.

[42] Tao J, Frankel AD (1992). Specific binding of arginine to TAR RNA. Proc Natl Acad Sci USA.

[43] Chenoweth DM, Dervan PB (2010). Structural basis for cyclic Py-Im polyamide allosteric inhibition of nuclear receptor binding. J Am Chem Soc.

[44] Abdel-Magid AF (2015). Allosteric modulators: an emerging concept in drug discovery. ACS Med Chem Lett.

[45] Munde M (2014). Structure-dependent inhibition of the ETS-family transcription factor PU.1 by novel heterocyclic diamidines. Nucleic Acids Res.

[46] Kopka ML, Yoon C, Goodsell D, Pjura P, Dickerson RE (1985). The molecular origin of DNA-drug specificity in netropsin and distamycin. Proc Natl Acad Sci USA.

[47] Tevis DS, Kumar A, Stephens CE, Boykin DW, Wilson WD (2009). Large, sequence-dependent effects on DNA conformation by minor groove binding compounds. Nucleic Acids Res.

[48] Hunt RA (2011). Induced topological changes in DNA complexes: influence of DNA sequences and small molecule structures. Nucleic Acids Res.

[49] Harteis S, Schneider S (2014). Making the bend: DNA tertiary structure and protein-DNA interactions. Int J Mol Sci.

[50] Wang AH (1979). Molecular structure of a left-handed double helical DNA fragment at atomic resolution. Nature.

[51] Jones RL, Lanier AC, Keel RA, Wilson WD (1980). The effect of ionic strength on DNA-ligand unwinding angles for acridine and quinoline derivatives. Nucleic Acids Res.

[52] Rocha MS, Cavalcante AG, Silva R, Ramos EB (2014). On the effects of intercalators in DNA condensation: a force spectroscopy and gel electrophoresis study. J Phys Chem B.

[53] Tiwari, N., Srivastava, A., Kundu, B. & Munde, M. Biophysical insight into the heparin-peptide interaction and its modulation by a small molecule. *J Mol Recognit* **31**, 10.1002/jmr.2674 (2018). 10.1002/jmr.2674 28961341

[54] Tanwar N, Munde M (2018). Thermodynamic and conformational analysis of the interaction between antibody binding proteins and IgG. Int J Biol Macromol.

