# Author Correction: A Comprehensive Biophysical Analysis of the Effect of DNA Binding Drugs on Protamine-induced DNA Condensation

**DOI:** 10.1038/s41598-020-66684-5

**Published:** 2020-06-09

**Authors:** Sakshi Gupta, Neha Tiwari, Manoj Munde

**Affiliations:** School of Physical Sciences, Jawaharlal Nehru University, New Delhi, 110067 India

Correction to: *Scientific Reports* 10.1038/s41598-019-41975-8, published online 10 April 2019

In the Supplementary Information file originally published with this Article, Supplementary Tables S1 and S2 were inadvertently switched. These errors have been corrected in the Supplementary Information that now accompanies the Article.

