# Supplementary material for: A Comprehensive Biophysical Analysis of the Effect of DNA Binding Drugs on Protamine-induced DNA Condensation
Source: Sci Rep. 2019 Apr 10;9:5891. doi: 10.1038/s41598-019-41975-8 (PMC6458161; doi:10.1038/s41598-019-41975-8)
Supplement: Supplementary file 1 — Supplementary information [file 41598_2019_41975_MOESM1_ESM.pdf]

Supporting Information

A Comprehensive Biophysical Analysis of the Effect of DNA Binding Drugs on Protamine-induced DNA Condensation

Sakshi Gupta[§], Neha Tiwari[§], Manoj Munde[§]*

[§]School of Physical Sciences, Jawaharlal Nehru University, New Delhi-110067, India

Contents

Figure S1. Plot of intensity of ^{CT}DNA obtained from gel electrophoresis band vs protamine/DNA molar ratio..S2

Figure S2. (A) UV melting profiles of ^{CT}DNA; protamine with ^{CT}DNA; ^{CT}DNA with EtBr; ternary complex of ^{CT}DNA with EtBr and protamine; ^{CT}DNA with Net; and ternary complex of ^{CT}DNA, Net and protamine and (B) data showing melting temperatures (T_m) for various ^{CT}DNA samples. The concentration of ^{CT}DNA was (50 µM), protamine (3 µM), EtBr (50 µM) and Net (25 µM)………………………………………………….....…………………….…... S2

Figure S3. (A) Kinetic measurements of ^{CT}DNA condensates as a function of time, plotted at 260 nm in the absence of drug and in the presence of EtBr and Net……………………………………………………………………....…. S3

Figure S4: ITC Binding studies of protamine (PT) (80 µM) into ^{CT}DNA (100 µM/bp) + DAPI (60 µM). Upper panel shows the data of the sequential injection of protamine into DAPI bound ^{CT}DNA and the lower panel represents the integrated heat against molar ratio of Protamine/DAPI bound ^{CT}DNA………………………………………….S4

Table S1. Thermodynamic parameters for ^{GC}DNA…………………………... S5

Table S2. Thermodynamic parameters for ^{AT}DNA…………………………... S5

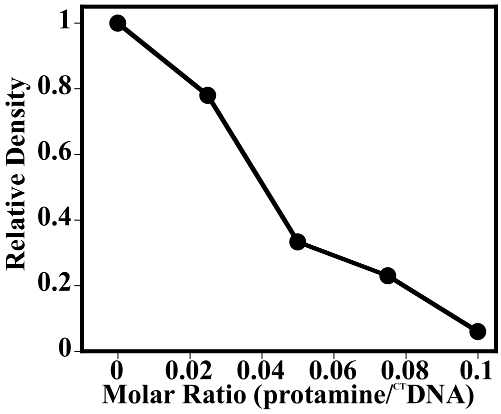

Figure S1. Plot of intensity of ^{CT}DNA obtained from gel electrophoresis band vs protamine/DNA molar ratio.

A

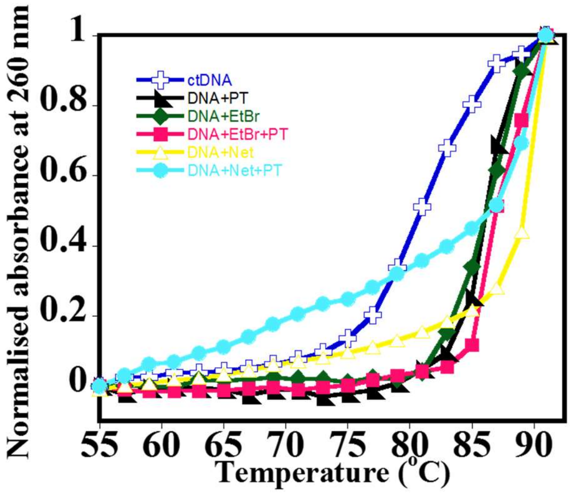

B

Sample	T_m (^{o}C)
^{CT}DNA	81
^{CT}DNA + Protamine	86
^{CT}DNA + EtBr	86
^{CT}DNA + Net	89
^{CT}DNA + EtBr + Protamine	87
^{CT}DNA + Net + Protamine	89

Figure S2. (A) UV melting profiles of ^{CT}DNA (+), protamine with ^{CT}DNA (◄), ^{CT}DNA with EtBr (♦), ternary complex of ^{CT}DNA with EtBr and protamine (■), ^{CT}DNA with Net (▲) and ternary complex of ^{CT}DNA, Net and protamine (●) and (B) data showing melting temperatures (T_m) for various ^{CT}DNA samples. The concentration of ^{CT}DNA was (50 μM), protamine (3 μM), EtBr (50 μM) and Net (25 μM). The melting studies of ^{CT}DNA provide the important information about its stability as a result of the addition of protamine or drug or both. The melting temperature (T_m) of ^{CT}DNA (81°C) was enhanced in the presence of protamine (87 °C), thus confirming the stabilization of ^{CT}DNA by 6 °C. The T_m of the ternary complex (^{CT}DNA +EtBr + protamine) showed similar enhancement (5 to 6 °C), suggesting competitive binding between protamine and EtBr with ^{CT}DNA. Net was also found to stabilize DNA in the similar fashion.

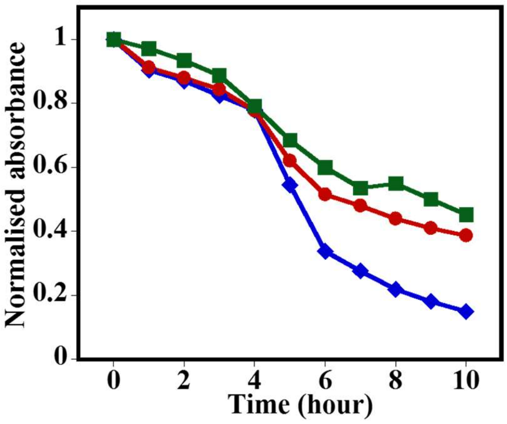

Figure S3. Effect of drugs on kinetics of intermolecular condensation (A) Kinetic measurements of ^{CT}DNA condensates as a function of time, plotted at 260 nm in the absence of drug (♦), in the presence of EtBr (●) and Net (■). Intramolecular condensation is too fast (32). However, intermolecular condensation takes place at larger timescale and can be studied using UV-vis as shown in Figure S3. The protamine-DNA complex showed a decrease in the absorbance with time at 260 nm, indicating that the DNA undergoes multimolecular complex formation which might be driven by DNA long-range interaction and crosslinking (22). However, the addition of Net or EtBr seemed to oppose the absorbance decrease, resisting the phenomenon of multimolecular condensation.

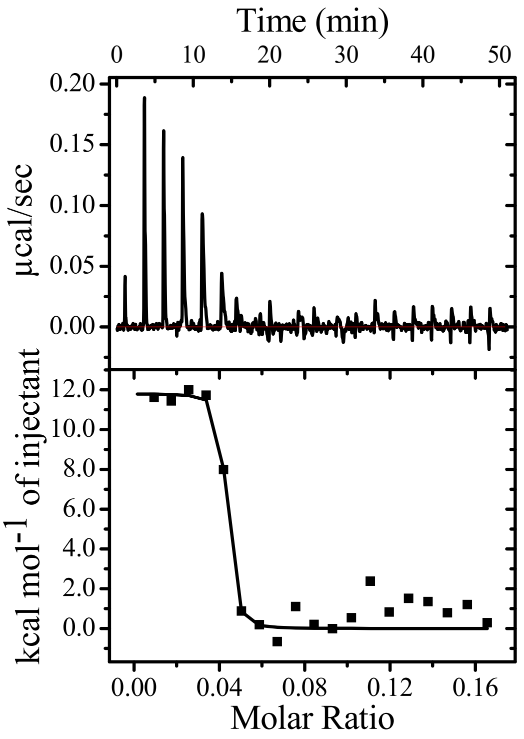

**K (M^{-1})**	1.6×10^8
**ΔH (kcal/mol)**	11.8
**TΔS (kcal/mol)**	23.0
**ΔG (kcal/mol)**	-11.2

Figure S4: ITC Binding studies of protamine (PT) (80 µM) into ^{CT}DNA (100 µM/bp) + DAPI (60 µM). Upper panel shows the data of the sequential injection of protamine into DAPI bound ^{CT}DNA and the lower panel represents the integrated heat against molar ratio of Protamine/DAPI bound ^{CT}DNA.

Table S1. Thermodynamic parameters for ^{GC}DNA.

Systems	N	K<br>(M^{-1})	ΔH<br>(kcal/mol)	TΔS<br>(kcal/mol)	ΔG<br>(kcal/mol)
**Protamine + ^{AT}DNA**	0.8 ± 0.005	$1.4x10^{8} \pm 5.7x10^{7}$	-15.1 ± 0.2	-4.0	-11.1
**Protamine + (EtBr-^{AT}DNA)**	0.7 ± 0.03	$3.9x10^{6} \pm 1.7x10^{6}$	-8.8 ± 0.5	0.1	-8.9
**EtBr + ^{AT}DNA**	4.3 ± 0.16	$1.65x10^{5} \pm 5.2x10^{4}$	-4.6 ± 0.2	2.44	-7.04
**Protamine + (Net-^{AT}DNA)**	0.7 ± 0.003	$1.89x10^{8} \pm 1.0x10^{8}$	-14.4 ± 0.15	-2.9	-11.3
**Net + ^{AT}DNA**	0.9 ± 0.015	$5.6x10^{7} \pm 3.5x10^{7}$	-6.8 ± 0.2	3.7	-10.5

Experiments were carried out in Hepes buffer at 25 °C. Errors for ΔH and N are fitting errors from ITC. Errors for K, ΔG, and TΔS are 15-20%.

Table S2. Thermodynamic parameters for ^{AT}DNA.

Experiments were carried out in Hepes buffer at 25 °C. Errors for ΔH and N are fitting errors from ITC. Errors for K, ΔG, and TΔS are 15-20%.

Systems	N	K<br>(M^{-1})	ΔH<br>(kcal/mol)	TΔS<br>(kcal/mol)	ΔG<br>(kcal/mol)
**Protamine + ^{GC}DNA**	1.4 ± 0.005	$3.4x10^{7}$	-17.5 ± 0.13	-7.3	-10.2
**Protamine + (EtBr-^{GC}DNA)**	0.7 ± 0.013	$4.5x10^{6}$	-15.7 ± 0.43	-6.7	-9.0
**EtBr + ^{GC}DNA**	3.2 ± 0.08	$1.45x10^{5}$	-9.8 ± 0.2	-2.8	-7.0
**Protamine + (Net-^{GC}DNA)**	0.75 ± 0.003	$4.5x10^{7}$	-17.29 ± 0.12	-6.9	-10.4
**Net + ^{GC}DNA**	-	-	-	-	-
